# Genetic Basis of Natural Variation in Spontaneous Grooming in *Drosophila melanogaster*

**DOI:** 10.1534/g3.120.401360

**Published:** 2020-07-29

**Authors:** Aya Yanagawa, Wen Huang, Akihiko Yamamoto, Ayako Wada-Katsumata, Coby Schal, Trudy F. C. Mackay

**Affiliations:** * Research Institute for Sustainable Humanosphere, Kyoto University, Uji, Japan; † Department of Animal Science, Michigan State University, East Lansing, Michigan 48824; ‡ Department of Entomology and Plant Pathology, North Carolina State University, Raleigh, NC 27695-7613; § Center for Human Genetics and Department of Genetics and Biochemistry, Clemson University, Greenwood, SC 29646

**Keywords:** Drosophila Genetic Reference Panel, genome wide association analysis, RNA interference, behavioral genetics

## Abstract

Spontaneous grooming behavior is a component of insect fitness. We quantified spontaneous grooming behavior in 201 sequenced lines of the *Drosophila melanogaster* Genetic Reference Panel and observed significant genetic variation in spontaneous grooming, with broad-sense heritabilities of 0.25 and 0.24 in females and males, respectively. Although grooming behavior is highly correlated between males and females, we observed significant sex by genotype interactions, indicating that the genetic basis of spontaneous grooming is partially distinct in the two sexes. We performed genome-wide association analyses of grooming behavior, and mapped 107 molecular polymorphisms associated with spontaneous grooming behavior, of which 73 were in or near 70 genes and 34 were over 1 kilobase from the nearest gene. The candidate genes were associated with a wide variety of gene ontology terms, and several of the candidate genes were significantly enriched in a genetic interaction network. We performed functional assessments of 29 candidate genes using RNA interference, and found that 11 affected spontaneous grooming behavior. The genes associated with natural variation in Drosophila grooming are involved with glutamate metabolism (*Gdh*) and transport (*Eaat*); interact genetically with (*CCKLR-17**D1*) or are in the same gene family as (*PGRP-LA*) genes previously implicated in grooming behavior; are involved in the development of the nervous system and other tissues; or regulate the Notch and Epidermal growth factor receptor signaling pathways. Several DGRP lines exhibited extreme grooming behavior. Excessive grooming behavior can serve as a model for repetitive behaviors diagnostic of several human neuropsychiatric diseases.

Animals perform various behaviors in response to their environment and internal state, including feeding, sound production, locomotion, mating, egg laying and self-grooming (Grillner and Wallén 2004; Grillner *et al.* 2005). Almost all animals, including insects, perform self-, mutual or allo-grooming behaviors. Grooming mechanisms have been studied in solitary insects using physical stimuli such as dust or tactile stimuli (Gratwick 1957; Hlavac 1975; Page and Matheson 2004). Aversive and microbial cues from the environment are important triggers of grooming behavior (Zhukovskaya *et al.* 2013; Yanagawa *et al.* 2017; 2019). In social insects, mutual grooming serves as a hygienic behavior (Yanagawa *et al.* 2008) and also distributes queen pheromone throughout the colony (Naumann 1991; Bahreini and Currie 2015). Insects also groom various body parts to remove noxious chemicals and maintain sensory acuity of the peripheral nervous system (Dethier 1972; Newland 1998; Page and Matheson 2004; Böröczky *et al.* 2013; Yanagawa *et al.* 2014). Recently, the output neural circuit of a mechanically induced grooming behavior, from the brain to motor neurons, has been described in *Drosophila melanogaster* (Seeds *et al.* 2014; Hampel *et al.* 2015; 2017). However, because grooming serves many functions and occurs spontaneously without extrinsic stimuli, the neural mechanisms that initiate and modulate spontaneous grooming behavior remain poorly understood.

Grooming is an innate behavior that follows a species-typical “stereotyped” sequence of actions by which animals clean themselves. The sequence of grooming behaviors is conserved across closely related species (Sachs 1988; Zhukovskaya *et al.* 2013), indicating that elements of this behavior are genetically based and important for survival. Indeed, mutational analyses and RNA interference (RNAi) have revealed several genes affecting grooming behavior in *D. melanogaster*, including *Hdc*, encoding *Histidine decarboxylase* (Melzig *et al.* 1996); the Drosophila ortholog of *Frm1*, which causes Fragile X Syndrome in humans (Tauber *et al.* 2011); *Dop1R1*, which encodes the D1 family dopamine receptor (Pitmon *et al.* 2016); *Nf1*, which encodes *Neurofibromin 1*, a Ras GTPase activating protein (King *et al.* 2016); *PGRP-LC*, which encodes *Peptidoglycan recognition protein LC*, which functions in immune response to bacteria (Yanagawa *et al.* 2017); as well as gustatory receptor genes (Yanagawa *et al.* 2019). However, little is known about the genetic basis of naturally occurring variation affecting the initiation and modulation of spontaneous grooming.

The *Drosophila melanogaster* Genetic Reference Panel (DGRP) is a population of wild-derived inbred fly lines with fully sequenced genomes, and is a community resource for genome wide association (GWA) mapping of variants associated with quantitative traits when all variants are known (Mackay *et al.* 2012; Huang *et al.* 2014). The DGRP harbors significant genetic variation for all quantitative traits assessed to date, including many behavioral traits (Mackay and Huang 2018). Here, we quantified the spontaneous grooming behavior of 201 DGRP lines and demonstrated that population variation in grooming time is genetically variable. We performed a GWA analysis of spontaneous grooming behavior and identified naturally occurring molecular polymorphisms associated with spontaneous grooming, and used RNA interference to functionally validate effects on grooming behavior for genes in which these polymorphisms were located. The candidate genes associated with spontaneous grooming are involved with neurotransmitter metabolism and transport; interact genetically with or are in the same gene family as genes previously implicated in grooming behavior; are involved in the development of the nervous system and other tissues; or regulate the Notch and Epidermal growth factor receptor signaling pathways.

## Materials and Methods

### Drosophila stocks

We used 201 DGRP lines that were generated and maintained at North Carolina State University (Mackay *et al.* 2012; Huang *et al.* 2014) to quantify grooming behavior and perform a GWA analysis for this trait.

We obtained *UAS*-RNAi lines for 29 candidate genes implicated by the GWA analysis from the Vienna Drosophila RNAi Center (VDRC): *CCKLR-17D3* (*P{KK110484}VIE-260B*, v102039); *CG13229* (*P{KK104316}VIE-260*, v100433); *CG17839* (*P{KK105048}VIE-260B*, v100149); *CG31221* (*P{KK113111}VIE-260B*, v103017); *CG33181* (*P{KK107703}VIE-260B*, v103142); *CG33926* (*P{KK112707}VIE-260B*; v107542); *CG34401* (*P{KK108172}VIE-260B*, v110636); *CG6767* (*P{KK107870}VIE-260B*, v109894); *Cht6* (*P{KK104493}VIE-260B*, v105536); *Eaat1* (*P{KK100187}VIE-260B*, v109401); *for* (*P{KK101298}VIE-260B*, v108293); *Gdh* (*P{KK107890}VIE-260B*, v109499); *GEFmeso* (*w^1118^ P{GD8852}v39952*, v39952), *kirre* (*P{KK107679}VIE-260B*, v109585); *Lrch* (*P{KK102050}VIE-260B*, v107047); *mtt* (*P{KK112979}VIE-260B*, v102982); *Nrg* (*P{KK100482}VIE-260B*, v107991); *osp* (*P{KK101464}VIE-260B*, v110701), *PGRP-LA* (*P{KK111407}VIE-260B*, v102277); *pnt* (*P{KK100473}VIE-260B*, v105390); *PsGEF* (*P{KK103731}VIE-260B*, v109769); *Ptp10D* (*P{KK101775}VIE-260B*, v110443); *Ptp99A* (*P{KK102836}VIE-260B*, v103931); *pxn* (*P{KK108816}VIE-260B*, v107180); *scat* (*P{KK100870}VIE-260B*, v108854); *SdhA* (*P{KK101728}VIE-260B*, v110440); *Smg5* (*P{KK102117}VIE-260B*, v107160); *sty* (*w^1118^*; *P{G**D1**156}v6948/TM3*, v6948); *Trf2* (*w^1118^*; *P{KK107538}VIE-260B*, v101318). The *P{KK}* RNAi lines are from the same genetic background and contain *UAS*-RNAi constructs for each candidate gene at the same locus on the second chromosome; the *P{GD}* RNAi lines are from the same genetic background, and contain *P*-element based transgenes in a random insertion site (Dietzl *et al.* 2007).

The control genotypes for the KK and GD RNAi lines are, respectively, *y,w^1118^*; *P{attP,y^+^,w^3^}* (v60100) and *w^1118^* (v60000), also obtained from the VDRC. We initially crossed all *UAS*-RNAi lines to an ubiquitously expressed *GAL4* driver, *Ubi-GAL4* (*P{Ubi-GAL4}2*). Lines without significant effects on grooming behavior using this driver were additionally crossed to two other ubiquitously expressed *GAL4* driver lines, *Act-GAL4* (*P{Act5C-GAL4}25FO1*) and *Ubi[156]-GAL4*. *Ubi-GAL4* and *Act-GAL4* were obtained from the Bloomington Drosophila Stock Center. *Ubi[156]-GAL4* was created in-house by introducing the original *Ubi-GAL4* transgene onto the third chromosome of the Canton-S B wild type strain by *Δ2-3* transposase-mediated hopping (Garlapow *et al.* 2015). All fly stocks were maintained at 25°.

### Behavioral assay

Spontaneous grooming of single-sex groups of 20 flies per sex and genotype was measured for each line for 3 min. All flies were mated and 3-5 days old at the time of the assays. The genotypes included the 201 DGRP lines, and the F1 offspring of *UAS*-RNAi and *GAL4*-driver parents. The F1 offspring of the *P{KK}* or *P{GD}* control line and the *GAL4*-driver parents are the controls for RNAi knock down. Flies were lightly anesthetized by placing them on ice for 3-5 min. They were then transferred singly to each well of 96-well plates (Cell Wells, 25858, Corning, USA). Thirty minutes after the transfer, the flies were video-recorded for three minutes. The duration of spontaneous grooming was quantified during the three minutes, separately for foreleg and hindleg grooming. The fly uses its forelegs to groom the eyes, head, thorax, forelegs and midlegs, and it uses the hindlegs to groom the hindlegs, genitalia and wings. The midlegs serve to groom the forelegs and hindlegs, so midleg grooming was included in foreleg grooming and hindleg grooming, respectively.

### Statistical analyses

We performed quantitative genetic analysis of total grooming time across sexes using the factorial mixed ANOVA model *Y* = *μ* + *Sex* + *Line* + *Sex* × *Line* + *e*, where *μ* is the overall mean, *Sex* is the fixed effect of sex, *Line* is the random effect of DGRP line, *Sex* × *Line* is the interaction between sex and genotype, and *e* is the residual. Reduced models were also fitted for the two sexes separately. We estimated the broad-sense heritability, $H^{2}$, as $H^{2}=\frac{\sigma_{L}^{2}+\sigma_{SL}^{2}}{\sigma_{L}^{2}+\sigma_{SL}^{2}+\sigma_{e}^{2}},$ where $\sigma_{L}^{2}$, $\sigma_{SL}^{2}$ and $\sigma_{e}^{2}$ are, respectively the among-line, sex by line and residual variance components. We estimated the cross-sex genetic correlation, $r_{MF}$, as $r_{MF}=\frac{\sigma_{L}^{2}}{\sigma_{L}^{2}+\sigma_{SL}^{2}}.$

We performed the GWA analysis of total grooming time using line means for each sex as described in Huang *et al.* (2014) and implemented at http://dgrp2.gnets.ncsu.edu. Briefly, we adjusted the phenotypic data for the effects of *Wolbachia* infection and major polymorphic inversions, and used the adjusted line means to fit a linear mixed model in the form of *y* = **X***b* + **Z***u + e*, where *y* is the adjusted phenotypic values, **X** is the design matrix for the fixed SNP effect *b*, **Z** is the incidence matrix for the random polygenic effect *u*, and *e* is the residual. The vector of polygenic effects *u* has a covariance matrix in the form of **A***σ*^2^, where *σ*^2^ is the polygenic variance component.

We annotated DNA variants using the gene models in Flybase release r5.57 (McQuilton *et al.* 2012). We downloaded the complete genetic interaction networks from FlyBase (release r5.57), where the genes are indicated by network nodes and the interactions between them as network edges. We mapped candidate genes from the GWA analysis (*P* < 10^−5^) to the network graph as described previously (Morozova *et al.* 2018). We extracted subnetworks from the global network whose edges were either a direct connection between candidate genes or bridged by only one gene not among the candidate gene list. We evaluated the significance of the size of the largest cluster among the subnetworks by a randomization test in which we randomly extracted subnetworks with the same number of input genes. The *P*-value was determined by dividing the number of instances where the size of the largest cluster exceeds the observed largest size by the total number of randomizations (*α*=0.05) (Antonov *et al.* 2010).

We used simple *t*-tests to assess differences in grooming behavior between the RNAi knock-down flies and control flies, separately for males and females. We also used Dunnett’s *t*-tests to correct for multiple testing and control the family-wise error rate.

### Data availability

The DGRP lines and the *Ubi-GAL4* and *Act-GAL4* drivers are publicly available from the Bloomington Drosophila stock Center at Bloomington, Indiana. All RNAi lines are publicly available from the Vienna Drosophila RNAi Center in Vienna, Austria. The *Ubi[156]-GAL4* driver is available upon request. All raw data and codes used have been uploaded to the GitHub repository https://github.com/qgg-lab/dgrp-grooming. Supplementary Table 1 gives the ANOVA of spontaneous grooming behavior and estimates of quantitative genetic parameters. Supplementary Table 2 gives the results of the GWA analyses of spontaneous grooming behavior, including SNP ID numbers and locations. Supplementary Table 3 provides the names and gene ontology annotations of significant genes from the GWA analyses. Supplementary Table 4 provides the results of the RNAi functional assessment analyses. File S1 is a short video of males and females from a high grooming line, DGRP_818. File S2 is a short video of males and females from a low grooming line, DGRP_517. Full DGRP sequence data are available at the NCBI Sequence Read Archive (SRA; http://www.ncbi.nlm.nih.gov/sra) under accession numbers listed in Supplemental Data File S1 of Huang *et al.* 2014. All SNP genotypes, quality metrics and annotations are available at http://dgrp2.gnets.ncsu.edu. Supplemental material available at figshare: https://doi.org/10.25387/g3.12252719

## Results

### Quantitative genetics of spontaneous grooming behavior in the DGRP

**Figure 1:**
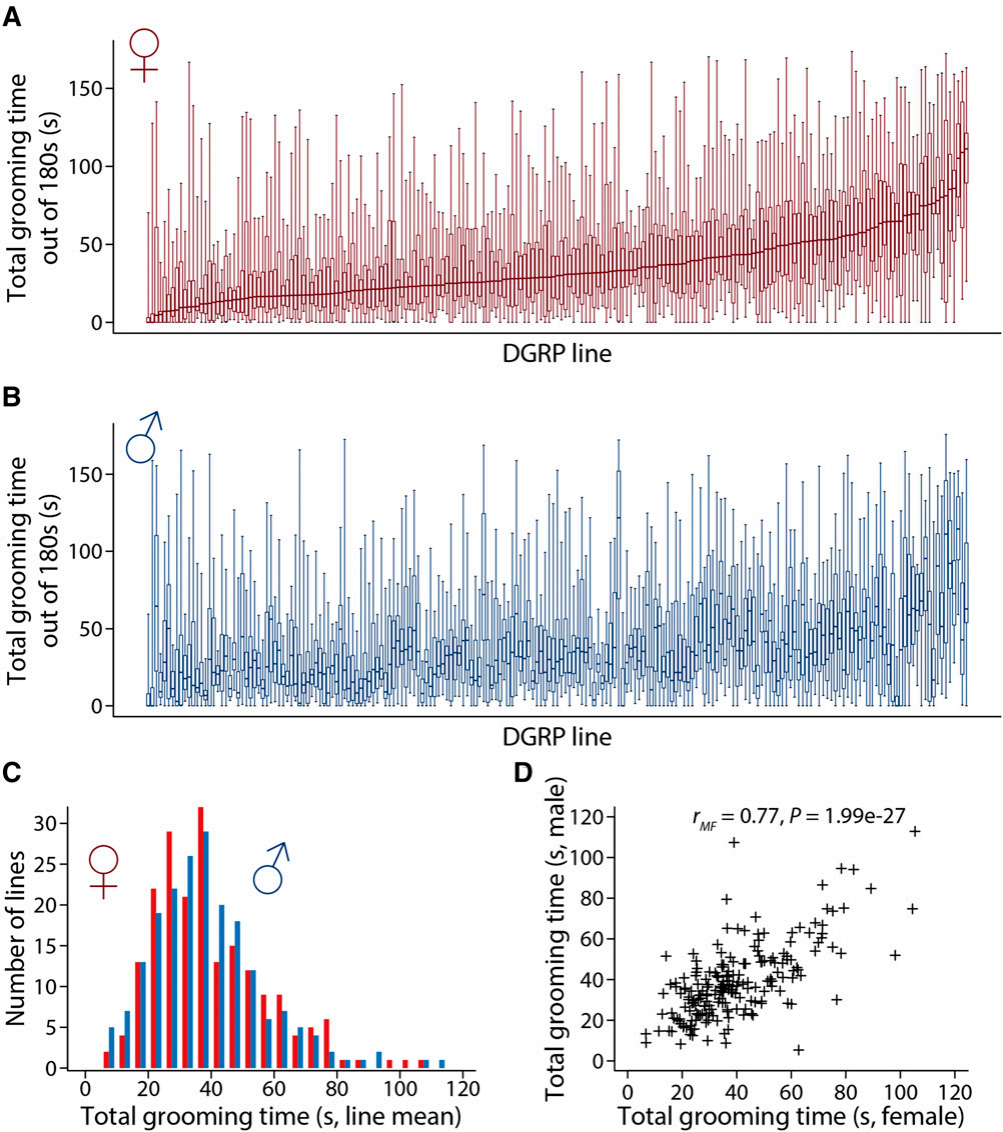
Variation in spontaneous grooming duration (s) in 201 DGRP lines. Flies were observed for 180 s. (a) Total grooming time for females, arranged from lowest to highest grooming time. The box plot for each line indicates the distributional property of the replicates. (b) Total grooming time for males. The lines are in the same order as for females. (c) Distribution of line means for grooming time in the DGRP. Lines are grouped in 5 s bins. The red histograms indicate female line means and the blue histograms indicate male line means. (d) Relationship between male and female grooming time for each line.

To understand the genetic basis of natural variation in spontaneous grooming behavior, we manually annotated video recordings of 20 females and 20 males from each of 201 DGRP lines, and quantified the total time spent grooming during a 3-minute period. There was substantial phenotypic variation both at the individual fly level (Figure 1a, b) and at the line level (Figure 1c) within each sex. Across all flies, spontaneous grooming time ranged from no grooming at all (0 s) to nearly constant grooming (176 s in a maximum of 180 s) (Files S1, S2). Across lines, the mean grooming time ranged from 6.7 s (DGRP_774) to 105 s (DGRP_805) in females, and from 5.4 s (DGRP_59) to 113 s (DGRP_805) in males. DGRP_805 was the most frequent groomer in both females and males (frequent grooming events result in longer cumulative grooming time). The heritability of total grooming time was 0.25 in females and 0.24 in males, with a cross-sex genetic correlation of 0.77 (Figure 1d), and significant genotype by sex interaction for this trait (Table S1). Thus, the genetic architecture of grooming behavior is partially in common and partially distinct between the sexes.

### GWA analysis for spontaneous grooming behavior

Because there was significant genetic variation for grooming behavior (Figure 1, Table S1), we performed a GWA analysis (Huang *et al.* 2014) to identify genes that harbor common (minor allele frequency, MAF > 0.05) DNA variants associated with variation in spontaneous grooming time (Table S2). We used line means for this analysis, which has the advantage of increasing the broad-sense heritability to *H*^2^ = 0.87, by reducing the residual variance by $\frac{\sigma_{e}^{2}}{n},$ where *n* is the number of individuals measured per sex and line. Only two variants were significant using a strict Bonferroni correction for nearly 2 million variants: *X*_8312234_INS in an intron of *Trf2* (*P* = 5.61 × 10^−9^ in males and *P* = 6.52 × 10^−9^ for both sexes) and the intergenic polymorphism *3L*_3396966_SNP (*P* = 2.18 × 10^−8^). At the more lenient significance threshold of *P* < 10^−5^, we identified 107 DNA variants associated with spontaneous grooming in either males or females, and/or the average or difference of the two sexes (Table S2). Of these variants, 34 were intergenic (> 1 kb from gene boundaries) while the remaining fell within 1 kb of 70 annotated genes (Table S2).

**Figure 2:**
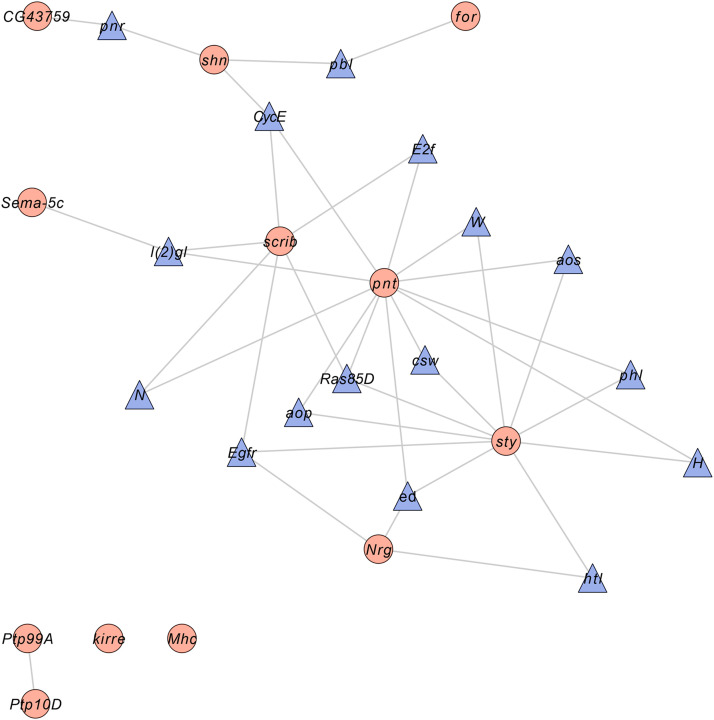
Genetic interaction network for spontaneous grooming behavior. The network consists of candidate genes identified by the GWA analysis at *P* < 10^−5^ (orange circles) and computationally recruited genes not identified in our study but which are known to interact with the candidate genes (purple triangles).

The genes associated with spontaneous grooming behavior fell into a wide variety of gene ontology (GO) categories, including 20 genes with GO terms associated with nervous system development and function; 11 genes with GO terms associated with organism or tissue development; seven genes associated with GO terms associated with transcriptional regulation; and seven genes with GO terms associated with epidermal growth factor, fibroblast growth factor, Notch, BMP or other signaling pathways (Table S3). We performed a GO enrichment analysis, accounting for difference in gene length as this affects the differential baseline probability of being identified in the GWA analysis. None of the tested GO terms were enriched for the grooming-associated genes, consistent with a highly polygenic genetic architecture. Next, we mapped the significant genes detected in the GWA analysis to known genetic interaction networks. We extracted subnetworks from the global network whose edges were either a direct connection between candidate genes or bridged by only one gene not among the candidate gene list, and evaluated the significance of the size of the largest cluster among the subnetworks by a randomization test in which we randomly extracted subnetworks with the same number of input genes. The subnetwork with no missing genes resulted in only two connected genes (*Ptp10D* and *Ptp99A*, *P* = 0.08). However, allowing genes to be connected by at most one gene not in the GWA gene list captured a cluster of 24 genes that was significantly larger than a randomly seeded network, centered on three highly connected genes: *pnt*, which encodes a transcriptional regulator; *scrib*, which encodes a synaptic scaffold protein; and the morphogenetic gene *sty* (*P* = 0.01, Figure 2).

### Functional assessment of candidate genes

**Figure 3:**
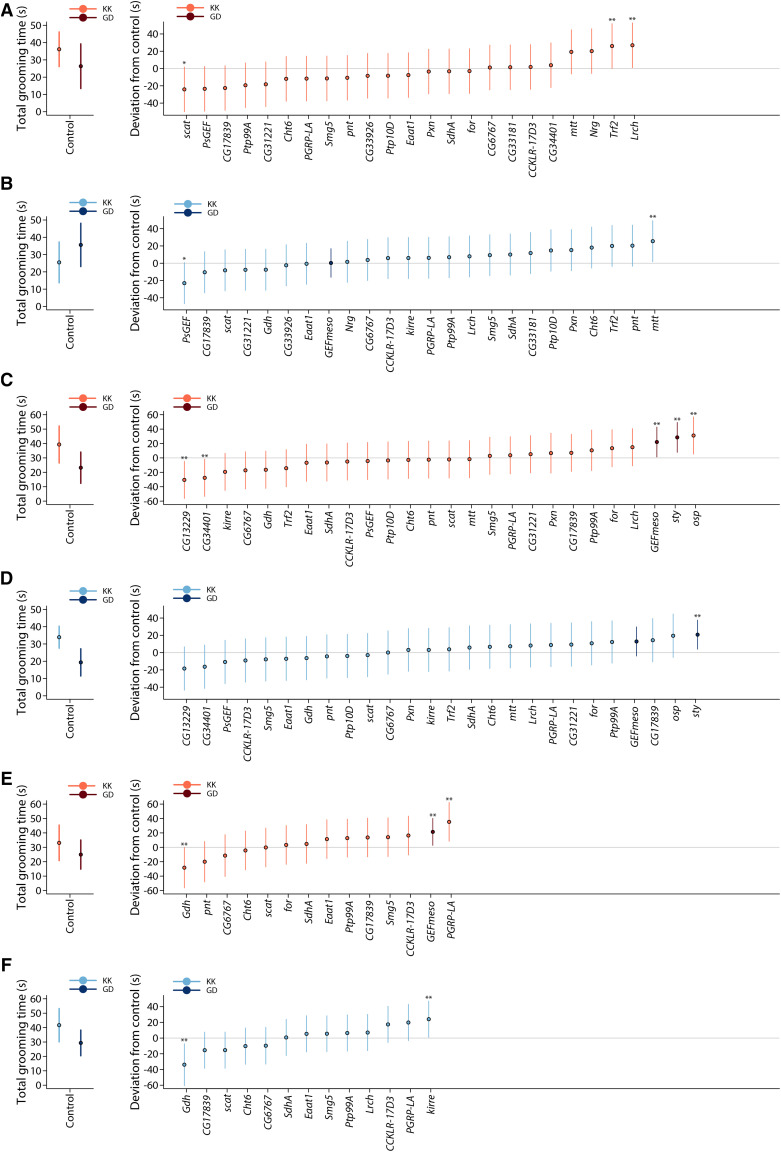
Results of RNAi functional assessments of candidate genes. The panels depict mean spontaneous grooming times (circles) for each of the tested *UAS*-RNAi/Driver-*GAL4* genotypes, expressed as a deviation from the appropriate Control/Driver-*GAL4* genotypes. The grooming times of the control genotypes are given to the left of each panel. The whiskers indicate the 95% Dunnett’s confidence intervals. (a) *UAS*-RNAi/*Ubi[156]-GAL4* females. (b) *UAS*-RNAi/*Ubi[156]-GAL4* males. (c) *UAS*-RNAi/*Ubi-GAL4* females. (d) *UAS*-RNAi/*Ubi-GAL4* males. (e) *UAS*-RNAi/*Act-GAL4* females. (f) *UAS*-RNAi/*Act-GAL4* males. * indicates *P* < 0.1 and ** indicates *P* < 0.05 from Dunnett’s tests.

We assessed whether ubiquitous reduction in gene expression of a sample of candidate genes implicated by the GWA analysis affected spontaneous grooming behavior, using the bipartite *GAL4/UAS*-RNAi system (Brand and Perrimon 1993; Dietzl *et al.* 2007). We chose 29 candidate genes that were most significant in males, females, or the difference between the sexes, and for which RNAi lines were available. We first crossed all 29 lines to an ubiquitous driver, *Ubi-GAL4*, and assessed spontaneous grooming behavior for RNAi lines producing viable adults. For lines that did not produce viable adults or that did not have a significant phenotypic effect when crossed to *Ubi-GAL4*, we crossed them to a weak ubiquitous driver (*Ubi[156]-GAL4*) and/or a strong ubiquitous driver (*Act-GAL4*). A total of 20 candidate genes had significant effects on spontaneous grooming behavior in at least one sex and driver combination at a nominal *P*-value of *P* < 0.05, and 11 genes had significant effects in the same direction in both sexes and/or for more than one driver (*CG13229*, *CG17839*, *CG34401*, *Gdh*, *GEFmeso*, *mtt*, *osp*, *PGRP-LA*, *PsGEF*, *sty*, *Trf2*) (Table S4). A total of 11 genes remained significant (*P* < 0.05) when we used Dunnett’s test to account for multiple tests when several treatments are compared to a control group (*CG13229*, *CG34401*, *Gdh*, *GEFmeso*, *kirre*, *Lrch*, *mtt*, *osp*, *PGRP-LA*, *sty*, *Trf2*) (Figure 3).

## Discussion

We have demonstrated that there is significant natural genetic variation in spontaneous grooming behavior in *D. melanogaster*, and that the genetic architecture of grooming behavior is partially distinct between males and females, as has been observed for most quantitative traits in the DGRP (Mackay and Huang 2018). We performed a GWA analysis and identified 107 molecular polymorphisms in 70 genes associated with spontaneous grooming behavior at a nominal *P* < 10^−5^. Only two of these polymorphisms resulted in nonsynonymous changes in protein coding genes; the rest presumably regulate gene expression, including the 34 polymorphisms located over 1,000 bp from the nearest protein coding gene.

None of the candidate genes identified in our GWA analysis has been associated previously with spontaneous grooming behavior, highlighting the complementary information to be gained from mutational analyses and naturally occurring variation due to common alleles. However, many of the candidate genes identified in this study are plausible given the trait assessed, and some are components of pathways implicated previously in grooming behavior. For example, glutamate is the primary excitatory neurotransmitter at the neuromuscular junction in insects (O’Kane 2011). *Gdh*, which encodes Glutamate dehydrogenase (Gdh); and *Eaat*, which encodes Excitatory amino acid transporter 1 (Eaat1), were candidate genes in the GWA analysis, and RNAi knockdown of *Gdh* affected grooming behavior. Gdh is involved in glutamate metabolism, and is the enzyme that catalyzes the reversible conversion of L-glutamate to α-ketoglutarate (Yelamanchi *et al.* 2016), while Eaat1 is a glutamate transporter (McQuilton *et al*. 2012).

*Nf1* is known to affect grooming behavior; *Nf1* genetically interacts with *CCKLR-17D1*, one of the candidate genes from the GWA analysis (Walker *et al.* 2013), as well as with *Ras85D* (Williams *et al.* 2001), which was computationally recruited to the significant genetic network derived from the GWA analysis candidate genes. The highly connected hub genes *scrib*, *pnt* and *sty* in the genetic interaction network also interact with *Ras85D*, suggesting Ras signaling may be involved in grooming behavior.

Grooming can be stimulated by mechanical (Page and Matheson 2004; Hlavac 1975; Gratwick 1957), olfactory (Becher *et al.* 2012; Stensmyr *et al.* 2012; Dweck *et al.* 2015; Kapsetaki *et al.* 2014), gustatory (Yanagawa *et al.* 2014; 2018; Soldano *et al.* 2016), and immunity-related (Yanagawa *et al.* 2014, 2017; Soldano *et al.* 2016) stimuli. We note that *scrib* (Ganguly *et al.* 2003) and *Sema-5c* (Sambandan *et al.* 2006; Rollmann *et al.* 2007) affect olfactory behavior. *PGRP-LC* has been associated with grooming behavior (Yanagawa *et al.* 2017); here, another candidate gene from this gene family, *PGRP-LA*, was a candidate gene from the GWA analysis, and RNAi knockdown of *PGRP-LA* affected grooming behavior. Peptidoglycan-recognition proteins regulate the immune deficiency pathway which controls the defense against Gram-negative bacteria (Gendrin *et al.* 2013).

The candidate genes associated with grooming behavior span a wide variety of gene ontology categories, suggesting novel genetic contributions to this complex behavior. Many of the genes are involved in nervous system development and/or function in organism or tissue development (including leg and muscle development, required for insect grooming) (Table S3). However, transcriptional regulation and post-transcriptional modification are also implicated. Finally, the Notch and Epidermal growth factor signaling pathways are implicated directly by the inclusion of *N* and *Egfr* as computationally recruited genes in the enriched genetic interaction network of candidate genes associated with spontaneous grooming behavior; and by the annotated biological function gene ontology categories of candidate genes in regulating Notch (*Kul*, *scrib*) and Epidermal growth factor (*kek5*, *pnt*, *Ptp10D*, *RhoGAP5A*, *scrib*, *sty*) pathway signaling (Table S3).

Functional assessment of 29 candidate genes using RNAi showed that 11 (*CG13229*, *CG34401*, *Gdh*, *GEFmeso*, *kirre*, *Lrch*, *mtt*, *osp*, *PGRP-LA*, *sty*, *Trf2*) affect spontaneous grooming behavior. However, the outcome of RNAi depends on many factors. RNAi can have off-target effects on assessed phenotypes (although few are predicted for the constructs utilized in this study), and ubiquitous RNAi affects all development times and tissues/cell types in which the target gene is expressed. It is possible that utilizing neuronal, glial or more specific *GAL4* drivers, or *GAL4* drivers with expression restricted to particular developmental stages, may have implicated more candidate genes, and future studies are needed to assess in which tissues/cell types and stages of development knockdown of expression affects the phenotype. Furthermore, RNAi cannot capture the potentially subtle effects of the naturally occurring variants; future variant-specific tests are needed for functional confirmation.

Grooming is an important fitness trait in insects (Yanagawa *et al.* 2008; 2017; 2019; Böröczky *et al.* 2013; Zhukovskaya *et al.* 2013; Yanagawa *et al.* 2014). Grooming is also a repetitive behavior. Aberrant repetitive behaviors are common for many human neurodevelopmental disorders (autism, fragile X syndrome, Prader-Willi syndrome, nonsyndromic intellectual disability) and clinical disorders (obsessive-compulsive disorder, Rett and Tourette syndrome, Parkinson’s disease, fronto-temporal and Alzheimer’s dementia) (Lewis and Kim 2009; Whitehouse and Lewis 2015). Further analysis of the genetic underpinnings of naturally occurring variation in Drosophila spontaneous grooming may yield insight into these human disorders.
